# Burrowing through the Heterogeneity: Review of Mouse Models of PTCL-NOS

**DOI:** 10.3389/fonc.2016.00206

**Published:** 2016-09-26

**Authors:** Christine E. Cutucache, Tyler A. Herek

**Affiliations:** ^1^University of Nebraska at Omaha, Omaha, NE, USA

**Keywords:** PTCL-NOS, T-cell lymphoma, mouse models, genetically engineered, neoplasms

## Abstract

Currently, there are 19 different peripheral T-cell lymphoma (PTCL) entities recognized by the World Health Organization; however, ~70% of PTCL diagnoses fall within one of three subtypes [i.e., peripheral T-cell lymphoma not otherwise specified (PTCL-NOS), anaplastic large-cell lymphoma, and angioimmunoblastic T-cell lymphoma]. PTCL-NOS is a grouping of extra-thymic neoplasms that represent a challenging and heterogeneous subset of non-Hodgkin’s lymphomas. Research into peripheral T-cell lymphomas has been cumbersome as the lack of defining cytogenetic, histological, and molecular features has stymied diagnosis and treatment of these diseases. Similarly, the lacks of genetically manipulated murine models that faithfully recapitulate disease characteristics were absent prior to the turn of the century. Herein, we review the literature concerning existing mouse models for PTLC-NOS, while paying particular attention to the etiology of this heterogeneous disease.

## Murine Models Recapitulating Clinical Disease Presentation of Mature T-cell Lymphomas: PTCL-NOS

T-cell malignancies can largely be classified into those affecting precursor T-cells and those affecting mature, post-thymic T-cells. Peripheral T-cell lymphomas (PTCLs) fall into the latter category and it is postulated that the cell of origin is an activated, memory cell – most likely a CD44+ T cell. Importantly, these are potentially cells with self-renewal capacity – a capacity typically only seen in stem cells, such as hematopoietic stem cells (1–6). There exists some overlap in terms of characteristics of PTCL-NOS and other T-cell lymphomas. For example, PTCL-NOS and angioimmunoblastic T-cell lymphoma (AITL) bear some similarities in terms of disease presentation, but PTCL-NOS is distinct from AITL in that AITL cells have solely a follicular T-helper phenotype (PD-1+, CXCL13+) and PTCL-NOS can include T-cells from differing subtypes (7). PTCLs express monoclonal T-cell receptor (TCR) or immunoglobulin rearrangement, as well as surface markers of both B- and T-cells (e.g., *BCL-1, BCL-2*, and *CD20*) (8–12). PTCLs are the most aggressive non-Hodgkin Lymphomas, exhibiting poor response to therapy, and poor prognosis with only 10–30% of patients having long-term survival (10, 13).

Therefore, the need to establish a model system to study this heterogeneous lymphoma subgroup is warranted. We have included solely genetically manipulated mouse models, as opposed to including xenograft models in immunocompromised mice, to study the impact of disease progression on the lymphoid compartment as part of the tumor microenvironmental niche. Therefore, we excluded models that were created without the influence of the immune response, as well as those where the B- or T-cell lymphoma were secondary to another disease driven by carcinogens or genetically engineered into the model. Similarly, this review does not include models, where PTCL-NOS is a secondary neoplasm or a “bystander” development. Therefore, this review includes the compilation of these models and the evidence for the potential etiology of PTCL-NOS.

While disease etiology can be exceedingly difficult to discern since tumors arise well before patients present with symptoms, murine models of mature T-cell lymphomas (a disease commonly understudied due to prevalence) are gaining ground because of their twofold ability to excellently recapitulate human disease and to point us in the direction of which cell of origin accounts for the different types of neoplasms. Of the two arms of lymphocytic lymphomas, B-cells have received the lion’s share of research to-date. For the largest type of mature T-cell lymphoma, peripheral T-cell lymphomas-not otherwise specified (PTCLs-NOS), three murine models really effectively model human disease. These models are the following: *Snf5*−*/*−, ITK–SYK fusion gene, and Lin28b.

Various types of animal models to recapitulate leukemias and lymphomas exist. The impact of such models to mimic lymphoproliferative diseases were reviewed previously (14). For this review, we conducted a literature search for murine models of PTCL-NOS. We conducted a literature search, a search of the NCI mouse database, Jackson Laboratory’s database, and MGI to identify all murine models for PTCL-NOS to-date. [A comprehensive summary of T-cell lymphoma murine models is provided by Warner et al. (15), and murine models of T-cell lymphoma, including transgenic, viral, carcinogen-induced, oncogene transformed, knockout, and mosaic models were last reviewed by Cook and Pardee (16)]. Prior reports of individual models capture the benefits and limitations of using mouse models to study lymphoma. Herein, we reviewed the models of PTCL-NOS in the last 15 years that mimic clinical presentation, disease severity, and genetic profiles common among patients.

### Loss of Tumor Suppressor Snf5 Leads to Aggressive PTCL

In an effort to recapitulate human PTCL, Wang et al. created *in vivo* models based on the tumor suppressor, *Snf5*, and determined the role of the TCR in tumorigenesis. *SNF5* is located at 22q11 and is deleted in 50% of T prolymphocytic leukemias, thereby suggesting its role as a tumor suppressor [(11, 17, 18); Table 1]. Specifically, a *Snf5*-deficient lymphoma cell line was generated by three sequential adoptive transfers of CD8+ splenocytes from *Lck-Cre* GFP+ Snf5^fl/fl^ tumor-bearing mice and was transplanted into sublethally irradiated C57BL/6J mice. *Snf5* inactivation in mice led to rapid onset PTCL (at ~11 weeks of age) (4, 11, 19). Mice with a functional TCR died at a mean age of 25 weeks, whereas those without a functional TCR (Rag−/−) lived past 50 weeks without any sign of tumor (11). CD4-Cre *Snf5^fl/fl^* and Lck-Cre *Snf5^fl/fl^* mice developed monoclonal or oligoclonal TdT−, TCR+, CD3+, CD8+, CD4− mature PTCLs in spleen, liver, and lymph node (though not thymus – since memory, rather than early stage T-cells were affected) (11). CD2-Cre *Snf5^fl/fl^* mice did not develop tumors (11). Furthermore, CD44 serves as a marker of memory phenotype and self-renewal capabilities on CD8+ memory T cells (3, 20). CD44^hi^ *Snf5*−*/*− cells were able to give rise to spontaneous tumors, whereas CD44^lo^ were unable to do so. These CD44^hi^ memory T cells were observed *before* tumor development, thereby implicating them as the potential cell of origin. Of total lymphoma cells in *Snf5* conditional knockout mice, 15% expressed the CD44^hi^ phenotype.

**Table 1:** **Summary of murine models of T-cell lymphomas**.

Gene manipulated	TS or oncogene	Chr(hum)	T-cell subtype affected	Functional pathway	% penetrance	Reference
*Snf5*−*/*−	TS	Ch22	Mature, activated memory	Self-renewal	100	(11,17, 18)
*ITK–SYK*	Oncogene	t(5;9)(q33;q22)^a^	Mature, activated memory	TCR signaling	100	(13)
*Lin28b*	Oncogene	6q21	Mature, activated memory	Let-7 miRNA signaling	100	(34)

*TS, tumor suppressor; Chr, chromosome; Hum, human*.

*^a^PTCL-NOS with follicular growth is observed in human cases with this translocation*.

Most importantly, Wang et al. aimed to determine whether tumorigenesis in this model was dependent upon TCR signaling (11). *Brg1* is part of the ATP-dependent chromatin remodeling complex SNF/SWI and regulates the expression of CD44. Specifically, loss of *Brg1* results in alterations in T cell development that is nearly identical to those caused by *Snf5*−*/*−, but lymphoma never forms from loss of *Brg1*. Therefore, there is a strong correlation between the enrichment of memory phenotype in the absence of *Snf5* and tumorigenesis. Consequently, to investigate tumorigenesis with TCR involvement, CD4-Cre *Brg1^fl/fl^* mice were used and did not give rise to CD44^hi^ memory T cells. Both Myc and stem cell-like profiles are enriched with loss of Snf5 and these are dependent upon TCR expression (11); specifically, Lck-Cre *Snf5^fl/fl^Rag2*−*/*− mice do not develop lymphoma. To that end, Eμ-tTA/tetO-MYC mice developed T cell lymphomas that can regress upon turning off Myc expression (21). Based on these data, tumorigenesis in a mature T-cell subtype is dependent upon TCR signaling (11). These data build upon other murine models with overexpression of the Myc transgene in hematopoietic cells – thus giving rise to both T-cell lymphomas and acute myeloid leukemias (21).

Moreover, after gene set enrichment analyses, Wang et al. identified 34 differentially expressed genes unique to CD44^hi^ cells that were downregulated in the non-tumorigenic CD44^lo^ population. Importantly, some of these genes (ID2, CD44, and CTLA2A) have already been identified, as playing critical roles in T-cell lymphomas (22–25), though previously their ability to contribute to the stem cell phenotype of these cancer cells was unknown. Therefore, *Snf5*−/− oncogenesis is dependent upon TCR signaling. These data shed light onto the role and impact of the TCR in T cell lymphoma.

### ITK–SYK Fusion Kinase Recapitulating PTCL

In human PTCL, the recurrent translocation 5(5;9)(q33;q22) results in a fusion kinase ITK–SYK that is inducible by interleukin-2. Additionally, 90% of PTCL cases have constitutive activation of SYK (26). The ITK–SYK complex associates with lipid rafts and triggers antigen-independent phosphorylation of proteins near the TCR, including CD69 (i.e., the phenotype of an activated T-cell) (13). SYK is part of the SYK/ZAP-70 family of non-receptor kinases modulating the TCR, Fc receptors on mast cells and macrophages, and BCR signaling (27–30). Moreover, SYK is present in immature T-cells and is downregulated upon selection of CD4+ and CD8+ cells (31–33). ITK and SYK are needed for APC: T cell interaction and subsequent T-cell activation.

Therefore, Pechloff et al. (13) created a *ITK–SYK* fusion kinase model that recapitulates PTCL. Interestingly, this model essentially functions to constitutively activate antigen–receptor expression or “mock TCR signaling,” and thereby drive PTCL. This suggests that the cells involved are antigen-experienced, and have an activated phenotype, consistent with human cases and further suggesting a cell of origin, even though ITK–SYK expression was detected in double positive T-cells during positive selection in the CD4-Cre *ITK–SYK* model (13) (Table 1).

The phenotype of the mice bearing this fusion oncogene mimicked that of clinical presentation of PTCL-NOS. Specifically, these mice had splenomegaly with ITK–SYK expressing TCR-β+ T-cells with an activated phenotype (CD44+), histologically the splenic architecture was abnormal and infiltrated by medium- to large-sized lymphocytes, and had increased cellular proliferation. The bone marrow in all animals was affected, and metastases were detected in kidney, liver, and lung and these were largely monoclonal, as assessed by TCR-Vβ analyses via flow cytometry and PCR (13). Interestingly, the expansion of lymphocytes was not limited to CD4; rather, 61% of animals had abnormal T cells that were CD4+, but 23% were CD8+, and the rest were double positive (CD4+ CD8+). Such a model may recapitulate extranodal T-cell lymphomas, such as subcutaneous panniculitis-like T cell lymphomas and enteropathy-associated T-cell lymphoma, best.

Similarly, Dierks et al. (30) created a murine model of PTCL using transplanted cells infected with a retrovirus carrying the ITK–SYK fusion gene (pMSCV ITK–SYK/IRES/GFP). Bone marrow of sublethally irradiated Balb/c mice was infected with either the fusion gene-containing vector or GFP-only vector. PTCL was observed infiltrating LNs, BM, and skin in under a month, systemic inflammation, increased IL-5 and IFNgamma production, and tumor cells (ITK–SYK+ T cells) were resistant to apoptosis. Interestingly, the disease was treatable with SYK inhibitors (though a search of current Clinical Trials at the time of writing this review produced no current trials using SYK inhibitors against lymphoma). This model was dissimilar to human presentation in that it lacked Bcl6 expression and CD10 was only in trace amounts (~10% of cells) (30). While, these mice did suffer from PTCL, the ability of this model to replicate human disease lacked in a few areas, as mentioned above. Moreover, these mice are laborious to maintain as compared with genetically engineered mice that can be bred subsequently to generate the phenotype in next generations.

### Transgenic Mice Overexpressing Lin28b Develop PTCL-NOS

*LIN28B, a master regulator of cellular transformation*, is commonly upregulated in PTCL ~7.5-fold to that of control, activated CD4+ cells (34) and controls the let-7 family of microRNAs. Hematopoietic compartment-specific, *Lin28b* transgenic mice have a similar disease profile to that of human PTCL. Specifically, infiltration of parenchymal organs with CD4+ tumor cells, inflammation, increased C-reactive protein, release of inflammatory cytokines, pleural effusion, and peripheral blood lymphopenia. The lymphopenia involved both CD4+ and CD8+ cells, with an expansion of tumorigenic CD4+ and CD8+ activated CD44+ CD62L− memory cells. These mice developed lymphoma at ~10 months of age – significantly longer than other models described herein. Similarly to human PTCLs, these tumor cells overexpressed SYK, had decreased let-7 expression, increased IL-6 expression, activation of the NF-κB pathway, infiltration by B-cells, and lymphadenopathy (34) (Table 1).

## Other Models of PTCL-NOS to Study Impact on CD8+ Cells or Developmental Questions

Other models that demonstrate T-cell proliferation, but at varying stages of cellular maturity, include the C3H/He-Tg(LCKprBCL2)36Sjk/J and B6;129S1-*Smarcb1^tm3Sho^/J* models. Specifically, the C3H/He-Tg(LCKprBCL2)36Sjk/J mouse includes the human LCKprBCL2 transgene, which leads to an increased percentage of cytotoxic T cells after 10 weeks of age and mice show robust survival following radiation treatment or with treatment of an anti-CD3 monoclonal antibody (35).

Another model on the B6 and 129 background has a similar timeline of disease development. The B6;129S1-*Smarcb1^tm3Sho^/J* strain has loxP sites in opposing orientation flanking the targeted gene with a reversible Cre-mediated conditional null allele. When crossed with Mx-Cre mice, these mice develop T-cell lymphoma with early onset of ~11 weeks of age in 100% of mice (19). Furthermore, *Snf5^inv/−^* mice developed the clinical presentation and histology that matches mature T-cell lymphoma. Interestingly, complete knockout of *Snf5* (or other members of the SWI/SNF chromatin remodeling complex) is embryonic lethal, though a single allele present leads to aggressive T-cell lymphoma very quickly (19).

Finally, the CALM/AF10 fusion gene is found in T-cell acute lymphoblastic leukemia and malignant lymphoma (as well as acute myeloid leukemia). The Vav-Cre-CALM/AF10-Tg model that has pan hematopoietic expression of the transgene exhibit lymphoma symptoms at 1 year of age, whereas no leukemia developed when the transgene was directed solely to CD19+ B-cells (36). Dutta et al. suggest that the cell of origin for CALM/AF10+ leukemias is a stem cell or early progenitor that acquires additional activation mutations leading to tumorigenesis.

While, these models recapitulate aggressive T-cell lymphomas and they have provided critical insight into T-cell biology (and etiology leading to PTCL), they are more helpful for studying developmental questions related to PTCL-NOS as compared with disease progression and potential treatment modalities, which represent the lionshare of work using murine models for PTCL-NOS.

## Summary

Often mouse xenograft models are used to mimic human disease, though these are poor models for T-cell lymphomas since the xenografts do not grow well in mice due to the lack of tumor microenvironment to sustain tumorigenesis. Therefore, either conditional knockouts or transgenic mice must be used in order to mimic T-cell lymphoma to study the biological consequences and eventually determine therapeutic applications useful for the disease.

While xenograft models for T-cell lymphomas have been difficult to create, Pechloff et al. and Wang et al. demonstrate that the cells of the models were transplantable into healthy animals with the original disease recapitulated. Additionally, the expansion of T-cells observed in all of these models was unique to malignant T-cells, and led to a decrease in overall healthy T-cells. These data suggest that the tumor cells replicate at the expense of healthy T cells, thereby suppressing the immune response and skewing it toward a more regulatory phenotype.

While all of the models described herein focused on disease recapitulation through manipulation of a tumor suppressor or oncogene, another model of aggressive T-cell lymphomas was created through chronic, low-dose lipopolysaccharide, and fed an alcohol-containing diet (37). Therefore, despite there being models centered around genetic mechanisms to drive T-cell lymphomas, it is very likely that epigenetic factors play a role as well. Genetically engineered mice prone to T-cell lymphoma such Eμ-*tTA/tetO-MYC* (21) and Lck-Tert (38) have also been generated (37). Although these models do not resemble the human disease phenotype nor affect only lymphoid cells, as the other models described herein do – likely because Myc and telomerase are critical to cellular proliferation, but have no direct association with the TCR or TCR-signaling partners. NOTCH and MYC serve as potent oncogenes in both B- and T-cell malignancies. Additionally, MYC modulates NOTCH and vice versa due to miRNA regulation through miR-30a (39). Models with and without miR-30a result in diffuse large B-cell lymphoma and T-acute lymphoblastic leukemia (39), thus mimicking human cases. Therefore, further studies will inform as to the role of miRNAs in PTCL-NOS.

Moreover, a recent study identified mutations in CD28 (the TCR co-receptor) in PTCLs (40). These data, coupled with the role the TCR plays in T-cell lymphoma, warrant investigation of a conditional knockout of CD28 on PTCL development. The basic biology of CD28 knockout mice B6.129X1-*Cd28*^tm1Jmg/^Mmjax (41) includes inability to generate germinal centers in the spleen. Similarly, the CD28*^tm1Jmg^* mice show a reduction in cytokine levels and failure to proliferate in mixed lymphocyte culture (41).

As this review summarizes the murine models to recapitulate PTCL-NOS to-date, we identify useful models based on time to disease presentation, differing genetic manipulations to lead to disease, and resultant phenotypes that bear resemblance to that observed in humans suffering from PTCL. Moreover, a review of these models shed light on the potential etiology of PTCL – likely, this is a mature cell, either single or double positive, and it bears stem cell-like capabilities to regenerate, even upon passage into cell culture and following injection into healthy animals.

## Author Contributions

CC and TH drafted the work, revised it critically, and approved this final version for publication.

## Conflict of Interest Statement

The authors declare that the research was conducted in the absence of any commercial or financial relationships that could be construed as a potential conflict of interest.
